# Comparison of fixed braces and clear braces for malocclusion treatment

**DOI:** 10.1186/s12903-024-04469-2

**Published:** 2024-08-14

**Authors:** Fan Liu, Yanhong Wang, Danzeng Luopei, Xiaofu Qu, Lin Liu

**Affiliations:** 1 Department of Orthodontics, Dalian Stomatology Hospital, 935 Changjiang Road, Shahekou District, Dalian 116021, Dalian, Liaoning, China; 2 Department of Prosthodontics, Dalian Stomatology Hospital, Dalian 116021, Liaoning, China; 3 Department of Cariology and Endodontics, Dalian Stomatology Hospital, Dalian 116021, Liaoning, China

**Keywords:** Stomatology, Orthodontic brackets, Malocclusion, Orthodontics, Fixed appliance, Retrospective studies

## Abstract

**Background:**

To study and compare the effects of clear aligners without brackets and traditional fixed aligners in orthodontic treatment.

**Methods:**

The samples were collected from January 2022 to April 2023. The control group (*n* = 26) received orthodontic treatment using traditional fixed appliances. The research group (*n* = 20) received orthodontic treatment using the clear aligners without brackets. Compare the therapeutic effects and related evaluation indicators between two groups.

**Results:**

The total effective ratio was compared between the 2 groups, and the study group was greater(*P* < 0.05). After treatment, the detected values of the periodontal condition indicators (plaque index, debris index, and gingival bleeding index), serum inflammatory factors (CRP, IL-6 and TNF-α) of the two groups, were less than before, also were all less than the control group. (*P* < 0.05). After therapy, in comparison of the control group, the value of mastication efficiency, comfort and psychological evaluation, sleep indicators and the points of the four dimensions of life quality in the study group was greater, and the detection results were obviously greater than before(*P* < 0.05).

**Conclusion:**

In the orthodontic therapy of sufferers with malocclusion, compared with the traditional fixed appliance, the clear aligners without brackets can enhance the treatment effects, improve the periodontal condition and masticatory function, and reduce the inflammatory responses, so that patients can feel more comfortable, thereby improving their psychology, sleep and quality of life. In the future, with the continual advancement of technology and people’s pursuit of beauty, the application of clear aligners without brackets in orthodontic treatment will become more and more extensive. The continuous introduction of new materials and new technologies will further improve the effects and comfort of the clear aligners without brackets, reduce treatment time and discomfort, and also reduce patients’ resistance to aligners, bringing patients a better treatment experience.

## Background

Malocclusion is a common disease in stomatology. It mainly refers to the incongruity in the position and relationship of the patients’ teeth, dental arch, jaw, and craniofacial, which is mainly caused by the factors of gene and environment [1–3]. After the occurrence of malocclusion, patients may experience symptoms such as overcrowding of the teeth and jaw, accompanied by obvious oral discomfort, which brings pain to the patients. Severe cases require economic costs for treatment, putting certain financial pressure on the patients. It can also lead to the deterioration of chewing function in patients, affect their daily diet. The impact on their daily interpersonal and social interactions can harm their mental health and significantly reducing their quality of life [4–7]. For malocclusion, it is advocated to carry out active treatment for such patients clinically. The treatment method is mainly orthodontic treatment, mainly through the use of fixed appliances to exert external stress on the patients’ jaw, so that the misaligned jaw can be corrected, thereby promoting the restoration of the dental-jaw relationship to normal [8–10]. The fixed appliance is the main tool used in orthodontic treatment, and it is also the key to the effects of orthodontic treatment. There is no uniform standard for the choice of fixed appliance in clinical practice. In the past, the fixed appliances used in orthodontic treatment are mainly traditional fixed appliances, which can correct the misalignment of teeth and jaws to a certain extent, but patients are prone to periodontal tissue inflammation responses during orthodontic treatment, the improvement effect of periodontal condition is not ideal. In recent years, as a new type of fixed appliance, the clear aligners without brackets has the advantages such as good concealment, high comfort, and easy cleaning compared with traditional fixed appliances. The frequency of it in the orthodontic treatment has increased in the department of stomatology. In order to explore the role of this fixed appliance in the orthodontic treatment of patients with malocclusion, this study selected patients who were applied in the stomatology department of the hospital from January 2022 to April 2023. Two groups of patients with malocclusion, 26 cases and 20 cases respectively, were treated with traditional fixed aligners and clear aligners without brackets. A retrospective analysis was carried out in the two groups to compare the therapeutic effects of two kinds of fixed appliances in patients with orthodontic treatment.

## Methods

### General data

The samples were collected from January 2022 to April 2023. Among the sufferers with malocclusion who received orthodontic therapy in the Department of Stomatology of the hospital at this stage, 26 patients with malocclusion who received orthodontic therapy with traditional fixed appliances and 20 sufferers with malocclusion who received orthodontic therapy with clear aligners without brackets were used as study samples, and they were set as a control one and a study one respectively. A retrospective analysis was carried out on the clinical data of the 2 groups of sufferers. The sex distribution of the control one was 10 men and 16 women, the age was between 18 and 34 years old, and a mean age of (26.35 ± 5.02) years old. The sex distribution of the study one was 8 men and 12 women, aged between 19 and 36 years old, and a mean age of (26.68 ± 4.91) years old. The two data of gender and age in the general information were compared between the 2 groups, and the results obtained through statistical analysis showed that *P* > 0.05, which confirmed that the two groups matched in general information and the study was comparable.

Inclusion criteria: (1) The patients who were diagnosed as malocclusion by orthodontic examination. It belongs to Angle’s I class malocclusion, that is, the upper and lower first molars have a correct occlusal relationship, but mesiobuccal space of maxillary first molar on either side or on one side of the neutral jaw occludes in the position of the buccal groove of the mandibular first molar, and some dental abnormalities, such as underbite, tooth crowding, which do not require tooth extraction orthodontic treatment; (2) The patients who aged not less than 18 years old and not higher than 40 years old; (3) The patients who were conscious when seeing a doctor, voluntarily accepted and cooperated with orthodontic treatment, and completed orthodontic treatment lasting 6 months; (4) The patients with completely preserved clinical data.

Exclusion criteria: (1) The sufferers who were complicated with other stomatological diseases; (2) The sufferers with cognitive impairment; (3) The sufferers who were complicated with pernicious tumors; (4) The sufferers with a history of orthodontic treatment; (5) The patients who were lost to follow-up during orthodontic treatment and dropped out of the study; (6) The patients who were free of systemic diseases; (7) The patients who were free of allergies; (8) The patients who were not receiving medications.

### Study methods

The control group used Gemini MBT metal orthodontic brackets produced by 3 M Company in the United States for orthodontic treatment. First, supragingival cleaning was carried out, and then the traditional straight wire arch fixed aligners were selected, and continued to wear the aligners for 6 months.

The research group carried out orthodontic treatment with clear aligners without brackets. First, supragingival cleaning was carried out, and then the Invisalign clear aligners without brackets produced by Align Technology Company of the United States were selected. The aligners were given to patients with instructions to be worn 20–22 h per day and removed only for eating and tooth brushing and continued to wear the aligners for 6 months.

During the period of orthodontic treatment in the two groups, oral follow-up visits were required once a month, and the patients were instructed to pay attention to cleaning the oral cavity every day.

### Observation indicators

The total effective ratio between the 2 groups was compared, and the various indicators (such as periodontal condition, mastication efficiency, and serum inflammation factors and sleep status), various scores (such as comfort, psychology and life quality)were compared between the 2 groups at pre-therapy and post-therapy.

Efficacy: The clinical efficacy after therapy was evaluated based on the criteria in the “Diagnostic and Treatment Routine of Stomatology”. It is divided into marked effect: the patients’ teeth are tilted < 5°, twisted teeth are < 10°, the teeth are arranged neatly, and the anterior teeth, overbites, and dentition return to normal; effective: the patients’ teeth are tilted 5°~15°, the teeth are twisted 10°~30°, the arrangement of the teeth is basically neat, and the anterior teeth, overbite, and dentition are basically restored to normal; invalid: the patients’ dentition deformity has not been corrected, and the arrangement of the teeth is not correct, and the anterior teeth and overbite are not significantly improved. Clinical total effective rate = marked rate + effective rate.

Periodontal condition: (1) Likert 4-grade (0–3 points) scale was used for the evaluation of plaque index and debris index, and the score was directly proportional to the amount of periodontal plaque and debris. (2) Gingival bleeding index was mainly measured by periodontal probe during assessment. The periodontal probe was inserted 1 mm below the gingival margin, and the gingival bleeding was observed while sliding the probe. The point was between zero and five, the greater the point, the more serious the gingival bleeding.

Chewing efficiency: the patients were instructed to chew 2 g of peanuts, 20 times on one side, and the spit and the residues in the pits and grooves of the tooth surface were taken out after rinsing, distilled water was added, stirred thoroughly, sieved, dried, weighed, and chewing efficiency was calculated. Chewing efficiency = weight difference before and after chewing / weight before chewing.

Serum inflammatory factors: blood was collected from the elbow veins of patients in the morning on an empty stomach, and the collected blood samples were centrifuged at the speed of three thousand rpm for ten minutes. The serum was taken as test samples for inflammatory factors. Inflammatory factors include C-reactive protein (CRP), interleukin-6 (IL-6), tumor necrosis factor-α (TNF-α), CRP was detected by immunoturbidimetry, IL-6 and TNF-α were both applied Enzyme-linked immunosorbent assay.

Comfort: the patients’ comfort was evaluated, the evaluation tool was mainly the GCQ General Comfort Scale. The scale is scored from 1 to 4 for 28 items, and the all points were between 28 and 112. The greater the point, the greater the comfort.

Psychology: Psychological evaluation indicators usually include anxiety and depression. The evaluation tools were the Anxiety Self-evaluation Scale(SAS) and the Depression Self-evaluation Scale(SDS) compiled by Professor Zung. Each scale contains 20 questions, and greatest point of each question is 4 scores. The critical value of the anxiety scale is 50 scores, and the critical value of the depression scale is 53 scores. The SAS has been shown to have good internal consistency with a Cronbach’s alpha of 0.82, and the SDS has fair internal consistency, with a Cronbach’s alpha of 0.79 [11]. The highest score is set to 100 points. And the score was inversely proportional to the mental health, that is, the higher the score, the less optimistic the mental health.

Sleep: Monitoring work on the latency to fall asleep at night and the actual sleep duration in patients was carried out, and polysomnography was used to monitor the sleep data of sufferers at night. At the same time, the sleep quality of sufferers was scored at night, and the Pittsburgh Sleep Quality Index (PSQI) scale was used for evaluation. And the highest score is set at 21 scores. The PSQI has been shown to have good internal consistency with a Cronbach’s alpha of 0.77 [12]. The greater the point, the more serious the problems encountered during night sleep.

Quality of life score: WHO Quality of Life Instrument Brief version (WHOQOL-BREF) was selected as the assessment tool of life quality. The scale sets the maximum score of the four aspects of physiology, psychology, environment and social relationship to one hundred scores. The greater the point, the greater the life quality level.

### Statistical methods

SPSS 22.0 was used in the statistical analysis of the data, the χ^2^ test was selected for the comparison of count data, and the Matched samples t-test was selected for the comparison of measurement data, and *P* < 0.05 indicated that the distinction between the data had statistical significance.

## Results

### The total effective ratio compared between the 2 groups

**Fig. 1:**
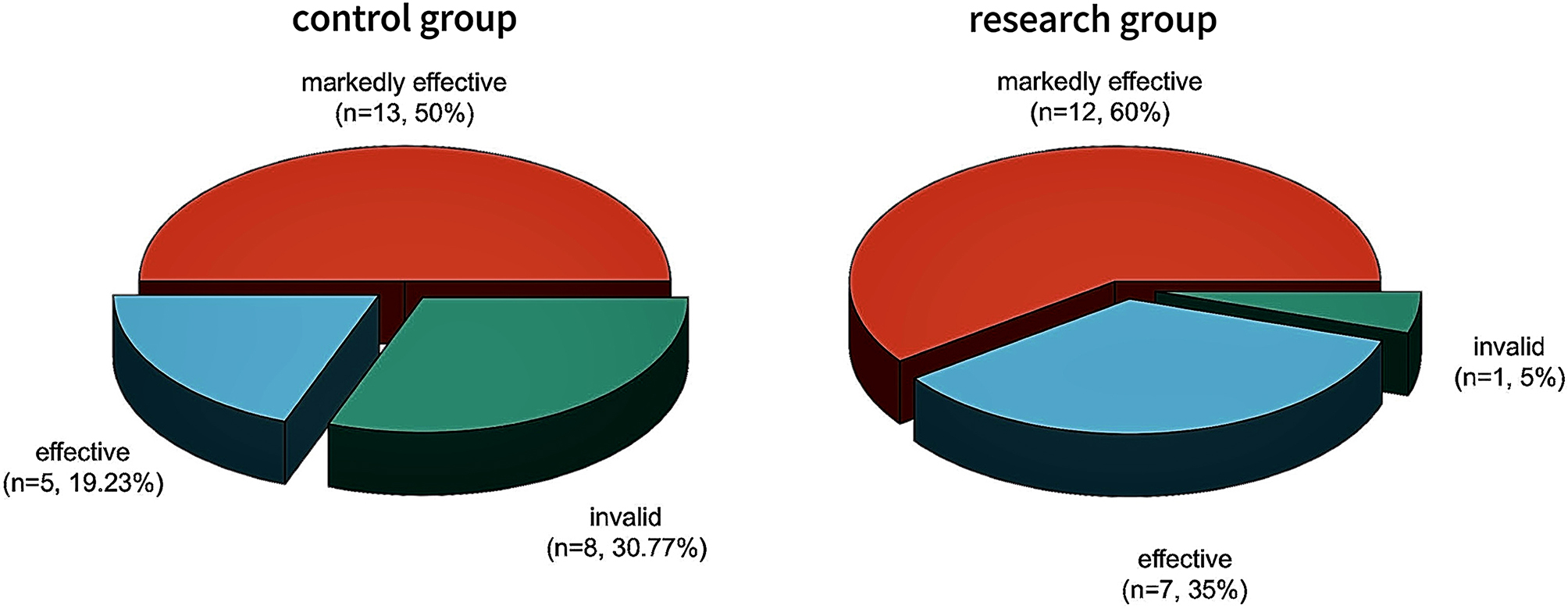
Histogram of clinical efficacy of the 2 groups

The total effective ratio was compared between the 2 groups, and the total effective ratio (95.00%) in the research one was greater than that in the control one (69.23%) (*P* < 0.05). See Table 1; Fig. 1.

**Table 1:** The total effective ratio compared between the 2 groups [n (%)]

Group	Number of cases	Markedly effective	Effective	Invalid	total effective ratio
Control group	26	13 (50.00%)	5 (19.23%)	8 (30.77%)	18 (69.23%)
Research group	20	12 (60.00%)	7 (35.00%)	1 (5.00%)	19 (95.00%) *

*Note* * means *P* < 0.05 compared with the control one

### The periodontal status indicators compared between the 2 groups

**Fig. 2:**
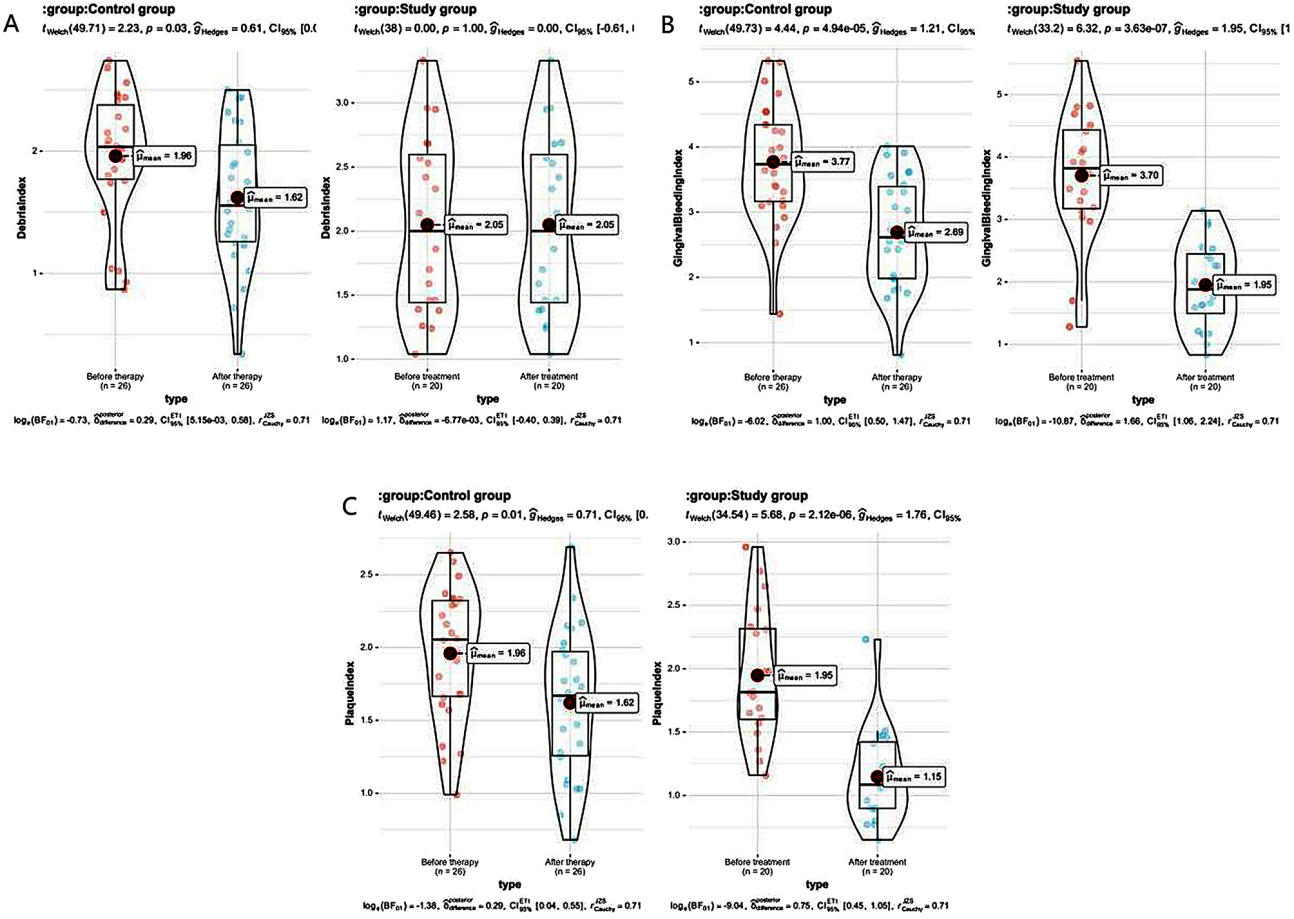
Histogram of periodontal status indicators in the two groups. *Note* * represents *P* < 0.05; ** represents *P* < 0.01

After therapy, the detected values of the three periodontal condition indicators of the two groups, namely the plaque index, debris index, and gingival bleeding index, were obviously less than those at pre-therapy, while the detection values of the three indicators in the study one were all less than those of the control one. (all *P* < 0.05). See Table 2; Fig. 2.

**Table 2:** The periodontal status indicators compared between the 2 groups ($\overline x \pm s$, points)

Group	Time	Plaque index	Debris index	Gingival bleeding index
Control group (*n* = 26)	Before therapy	1.96 ± 0. 45	1.96 ± 0. 53	3. 77 ± 0.91
	After therapy	1. 62 ± 0. 50 ^#^	1. 62 ± 0. 57 ^#^	2. 69 ± 0. 84 ^#^
Study group (*n* = 20)	Before treatment	1.95 ± 0. 51	2.0 5 ± 0. 69	3. 70 ± 1. 03
	After treatment	1.1 5 ± 0.3 7 ^#^ *	1. 20 ± 0. 41 ^#^*	1. 95 ± 0. 69 ^#^*

*Note*^#^ means *P* < 0.05 compared with before therapy, * means *P* < 0.05 compared with the control one

### The mastication efficiency compared between the 2 groups

**Fig. 3:**
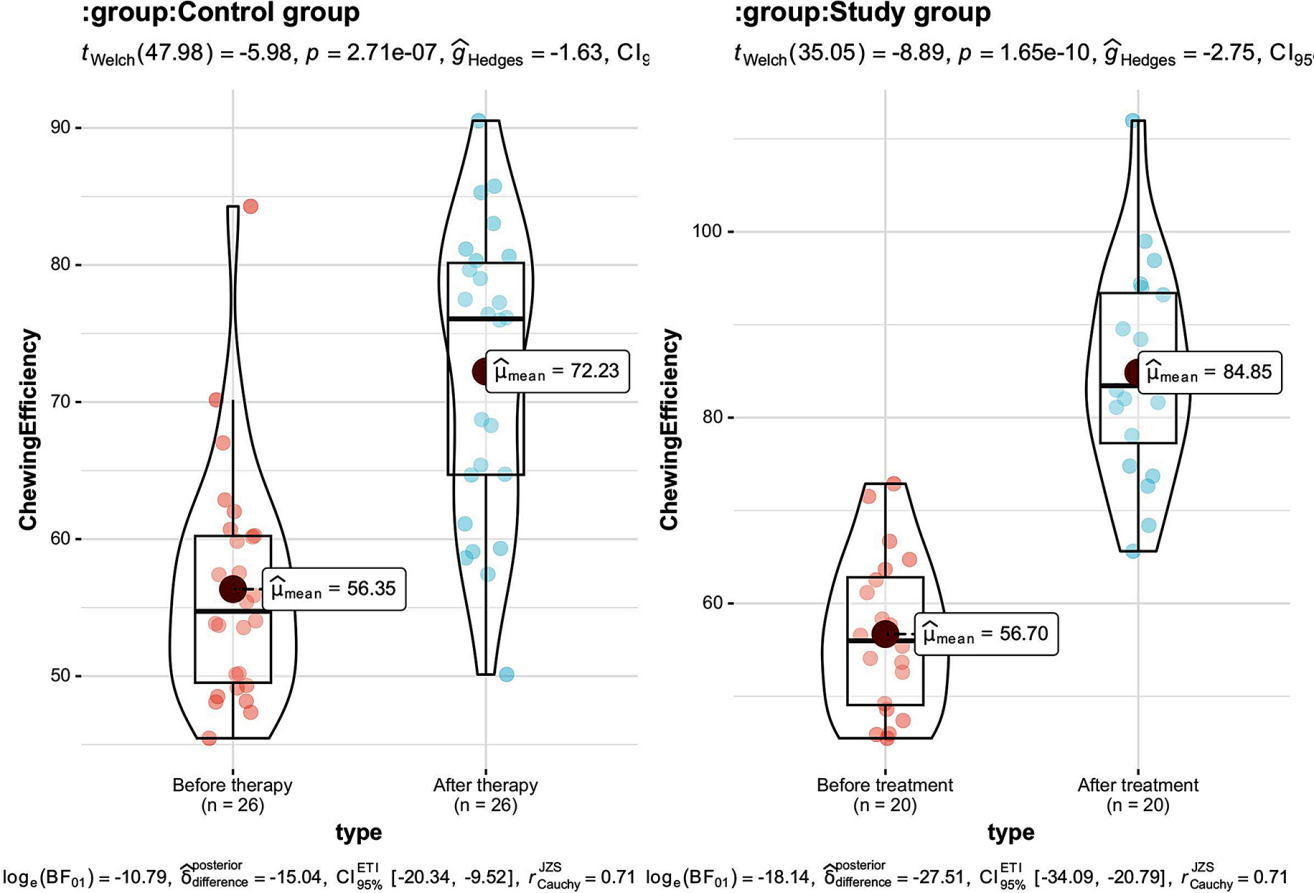
Histogram of mastication efficiency in two groups. *Note* *** stands for *P* < 0.001

After therapy, compared with the control one, the value of mastication efficiency in the study one was greater, and the detection results of mastication efficiency in the 2 groups were obviously greater than those before therapy (all *P* < 0.05). See Table 3; Fig. 3.

**Table 3:** Comparison of mastication efficiency between the 2 groups ($\overline x \pm s$, %)

Group	Time	Chewing efficiency
Control group (*n* = 26)	Before treatment	56.35 ± 8. 54
	After treatment	72. 23 ± 10 0.52 ^#^
Study group (*n* = 20)	Before treatment	56. 70 ± 8. 44
	After treatment	84. 85 ± 11. 37 ^#^*

*Note*^#^ means *P* < 0.05 compared with before therapy, * means *P* < 0.05 compared with the control one

### The serum inflammatory factor indicators compared between the 2 groups

**Fig. 4:**
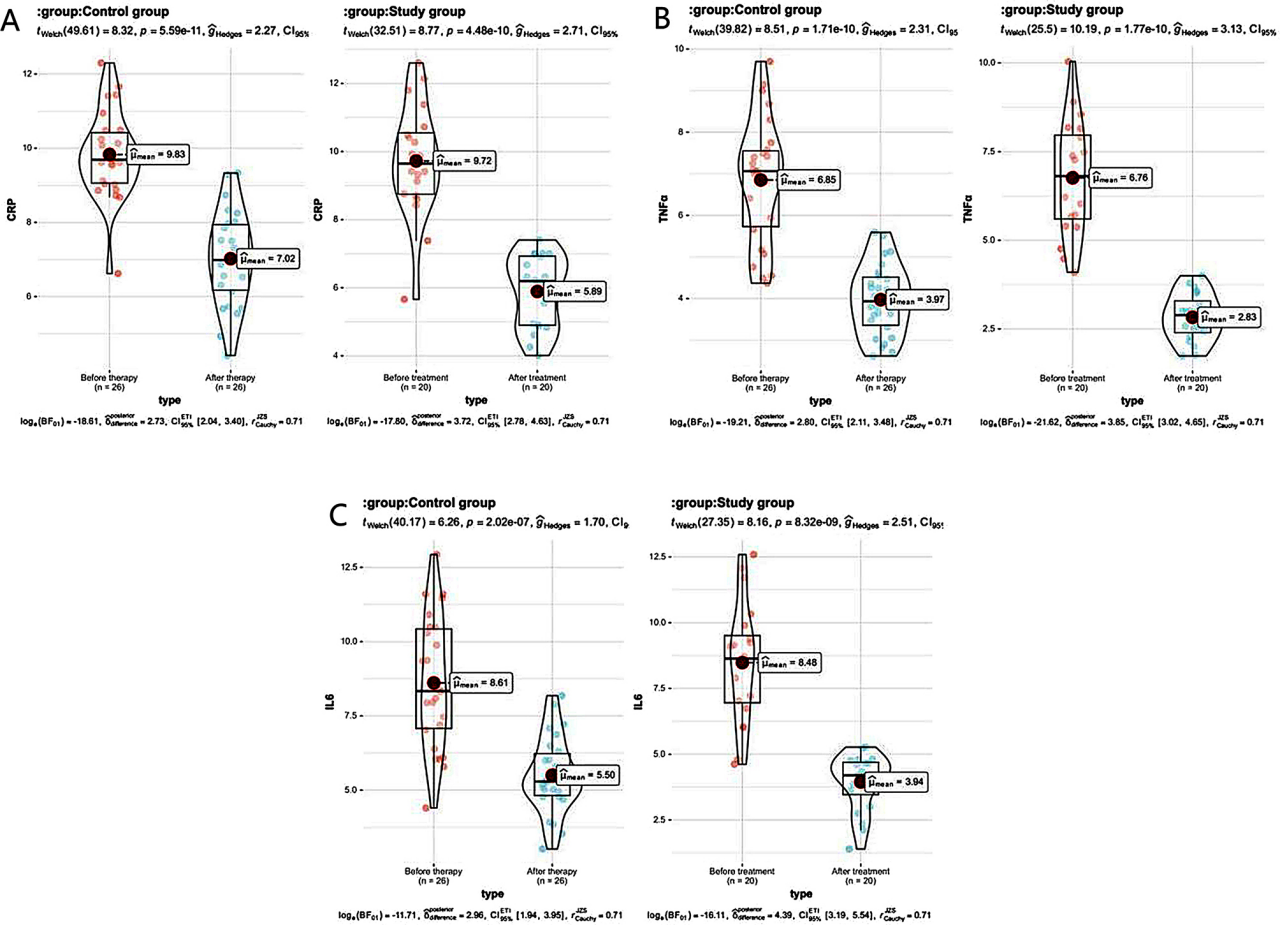
Histogram of serum inflammatory factor indicators in the 2 groups. *Note* ** represent *P* < 0.01; *** represented *P* < 0.001; **** represent *P* < 0.0001

After therapy, the detected values of serum inflammatory factors of CRP, IL-6 and TNF-α in the 2 groups were obviously less than those before therapy, while the detected values of the three serum inflammatory factors in the study one were less than the control one (all *P* < 0.05). See Table 4; Fig. 4.

**Table 4:** The serum inflammatory factor indicators compared between the 2 groups ($\overline x \pm s$)

Group	Time	CRP (mg/L)	IL-6 (pg / mL)	TNF-α (µg / L)
Control group (*n* = 26)	Before treatment	9.83 ± 1.61	8.61 ± 2.19 _ _ _	6.85 ± 1.50
	After treatment	7.02 ± 1.27 ^#^	5.50 ± 1. 27 ^#^	3.97 ± 0.86 ^#^
Study group (*n* = 20)	Before treatment	9.72 ± 1.64	8. 48 ± 2.24	6.76 ± 1.59
	After treatment	5.89 ± 1.06 ^#^ *	3.94 ± 1.08 ^#^*	2.83 ± 0.67 ^#^ *

*Note*^#^ means *P* < 0.05 compared with before therapy, * means *P* < 0.05 compared with the control one

### Comparison of comfort and psychological points between the 2 groups

**Fig. 5:**
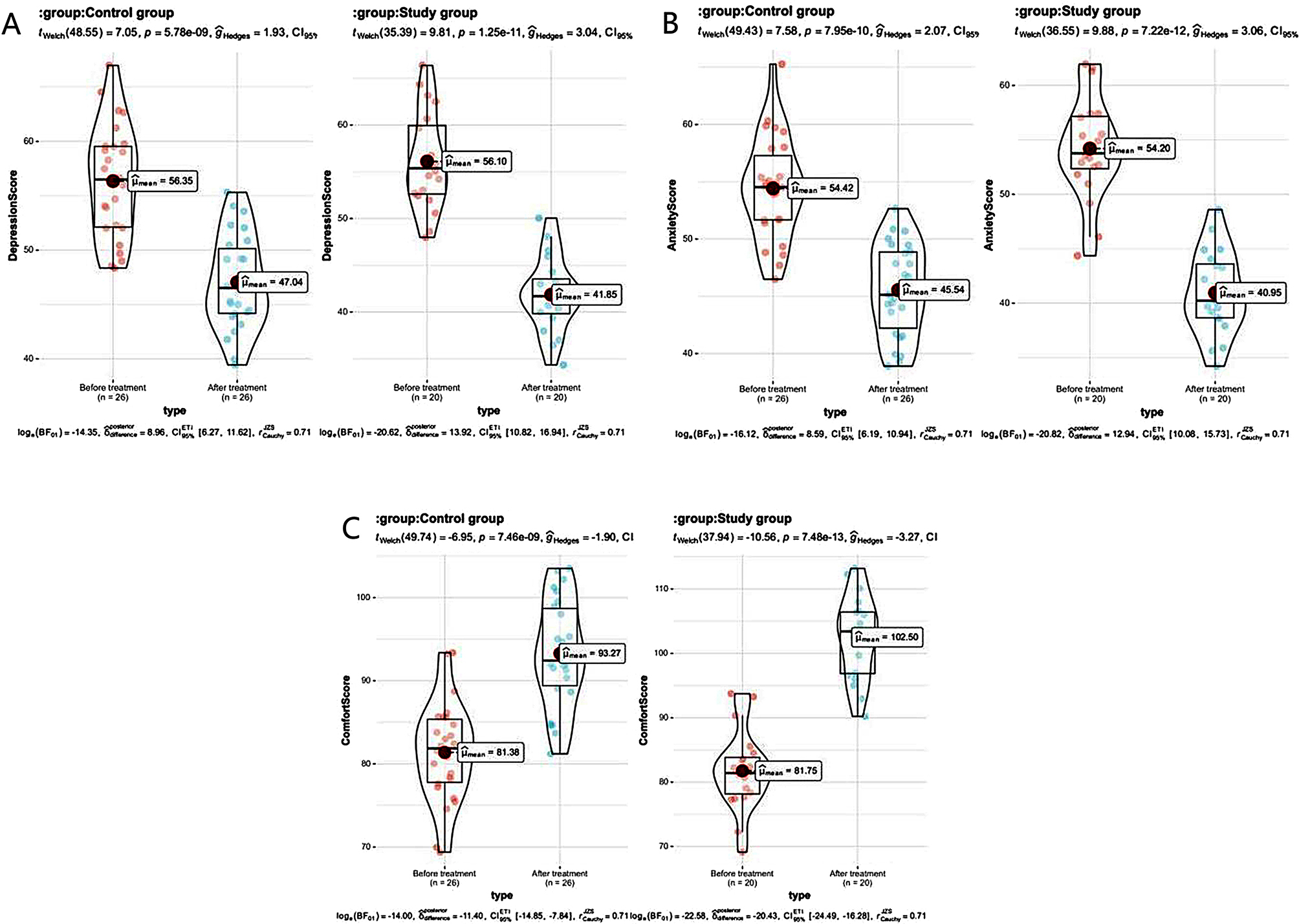
Histogram of comfort level and psychological scores of the 2 groups. *Note* *** represent *P* < 0.001; **** represents *P* < 0.0001

After therapy, the points of comfort and psychological evaluation of the 2 groups were obviously improved compared with those before therapy, while the score of the comfort evaluation of the research one was greater than the control one, and both points in the psychological evaluation were less than the control one (all *P* < 0.05). See Table 5; Fig. 5.

**Table 5:** The comfort and psychological points compared between the 2 groups ($\overline x \pm s$, points)

Group	Time	Comfort score	Anxiety score	Depression score
Control group (*n* = 26)	Before treatment	81. 38 ± 5. 95	54. 42 ± 4. 44	56. 35 ± 5. 15
	After treatment	93. 27 ± 6. 39 ^#^	45. 54 ± 3. 99 ^#^	47.04 ± 4. 33 ^#^
Study group (*n* = 20)	Before treatment	81. 75 ± 6. 09	54. 20 ± 4. 64	56. 10 ± 5. 18
	After treatment	102. 50 ± 6. 34 ^#^ *	40. 95 ± 3.79 ^#^ *	41 0.85 ± 3. 92 ^#^ *

*Note*^#^ means *P* < 0.05 compared with before therapy, * means *P* < 0.05 compared with the control one

### Comparison of sleep status indicators between the 2 groups

**Fig. 6:**
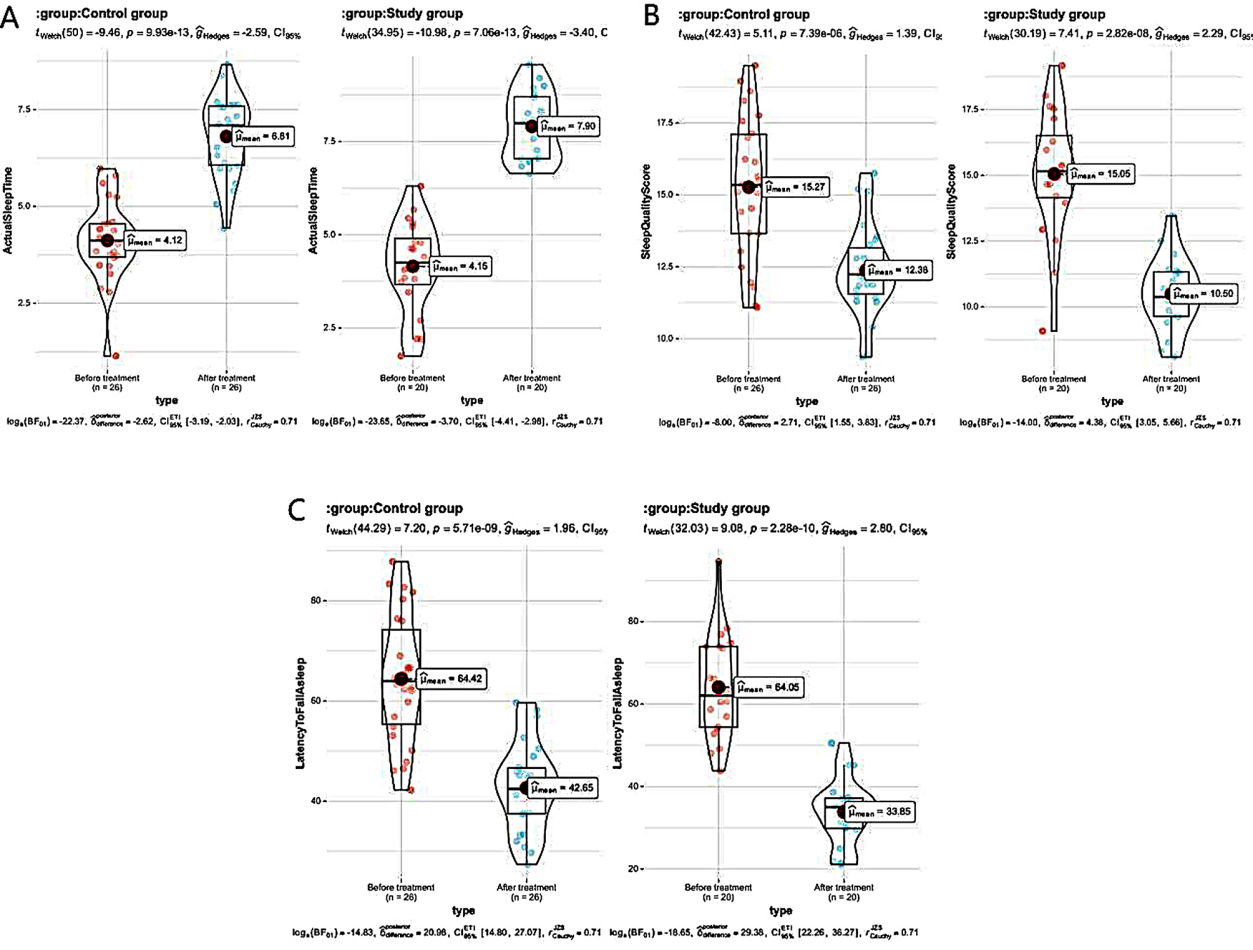
Histogram of sleep status indicators in the two groups. *Note* ** represent *P* < 0.01; *** represent *P* < 0.001

After therapy, the sleep indicators of the 2 groups at night monitoring and evaluation were significantly improved compared with those at pre-therapy, while in comparison of the control one, the research one had better values in latency to fall asleep and actual sleep duration, and lower score in assessment of sleep quality (all *P* < 0.05) See Table 6; Fig. 6.

**Table 6:** The sleep status indicators compared between the 2 groups ($\overline x \pm s$)

Group	Time	Latency to fall asleep (min)	Actual sleep time (h)	Sleep quality score (points)
Control group (*n* = 26)	Before treatment	64.42 ± 12.72	4.12 ± 1. 03	15.27 ± 2. 43
	After treatment	42.65 ± 8.73 ^#^	6. 81 ± 1.02 ^#^	12.3 8 ± 1.55 ^#^
Study group (*n* = 20)	Before treatment	64. 05 ± 12.59	4. 15 ± 1. 23	15. 05 ± 2. 39
	After treatment	33. 85 ± 7.93 ^#^ *	7.90 ± 0.91 ^#^ *	10. 50 ± 1.36 ^#^ *

*Note*^#^ means *P* < 0.05 compared with before therapy, * means *P* < 0.05 compared with the control one

### Comparison of life quality scores between the 2 groups

**Fig. 7:**
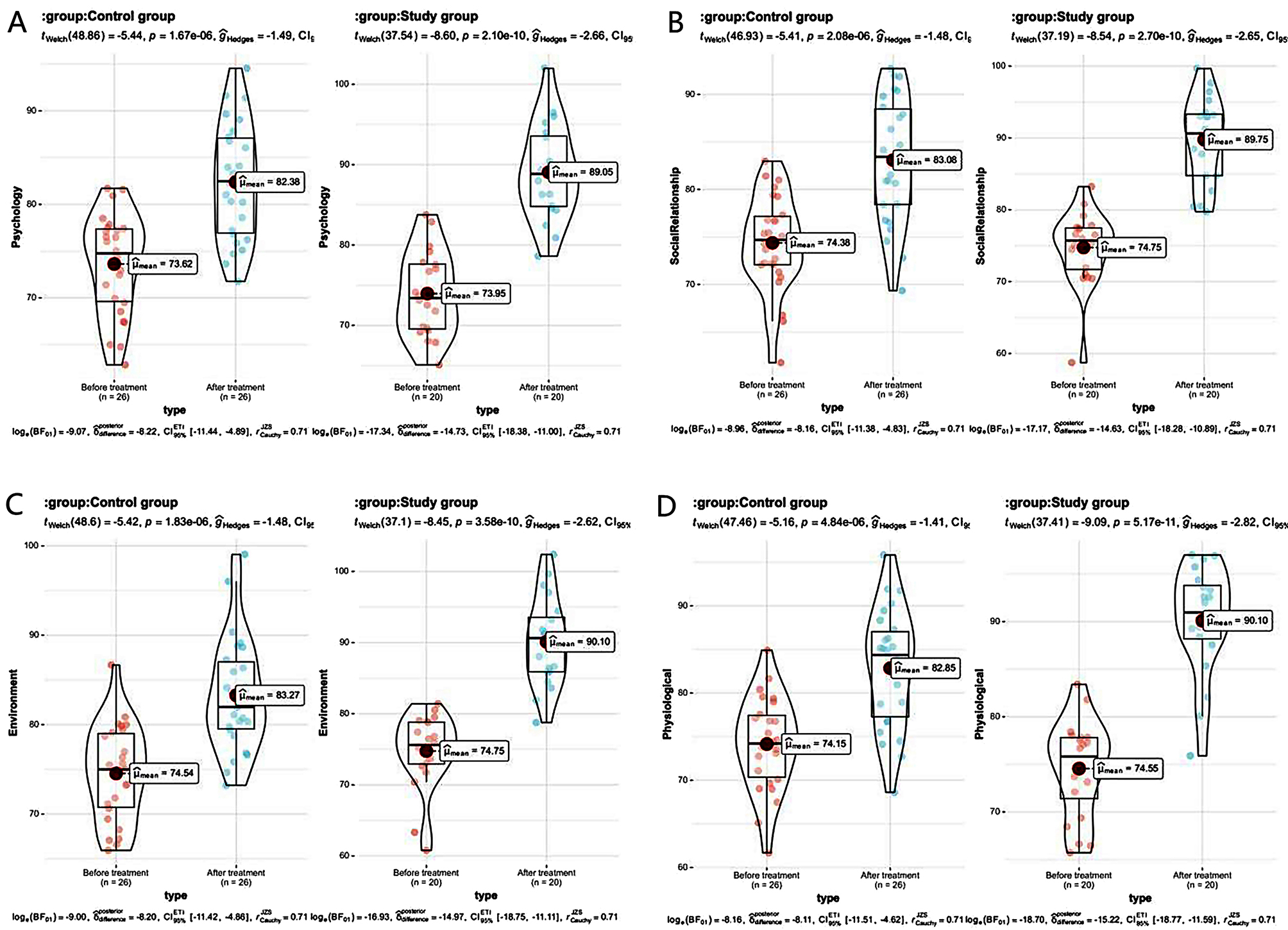
Histogram of life quality scores in the two groups. *Note* ** represent *P* < 0.01; *** represents *P* < 0.001

After therapy, the points of the four dimensions of life quality in the 2 groups all increased obviously, and the points of each dimension of life quality of the research one were greater than the control one (all *P* < 0.05). See Table 7; Fig. 7:

**Table 7:** The life quality points compared between the 2 groups ($\overline x \pm s$, points)

Group	Time	Physiological	Psychology	Environment	Social relationship
Control group (*n* = 26)	Before treatment	74.15 ± 5. 33	73. 62 ± 5. 34	74. 54 ± 5. 29	74. 38 ± 5. 00
	After treatment	82. 85 ± 6. 75 ^#^	82. 38 ± 6. 23 ^#^	83. 27 ± 6. 28 ^#^	83. 08 ± 6. 50 ^#^
Study group (*n* = 20)	Before treatment	7 4. 55 ± 5. 06	7 3. 95 ± 5. 23	7 4. 75 ± 5. 28	7 4. 75 ± 5. 13
	After treatment	90.10 ± 5.74 ^#^ *	89. 05 ± 5.85 ^#^ *	90. 10 ± 6. 18 ^#^ *	89.75 ± 5.95 ^#^ *

*Note*^#^ means *P* < 0.05 compared with before therapy, * means *P* < 0.05 compared with the control one

## Discussion

Malocclusion is a common disease in stomatology, which is mainly caused by the factors of congenital gene and acquired environment, such as bad oral habits, periodontal disease, trauma, etc [13–15]. . . Malocclusion is mainly distributed in the upper and lower dentition of the oral and maxillofacial region, which will lead to symptoms such as irregular dentition and misalignment of teeth, and abnormal relationship between the upper and lower dental arches, which will further affect the occlusal function of the patients and reduce the chewing efficiency. And there is a lot of inconvenience in the daily meal, which has a serious effect on the daily life of patients, resulting in a decline in their life quality [16–18]. And because malocclusion will affect the aesthetics of the patients’ oral cavity, it may also affect the patients’ maxillofacial morphology, and the appearance of the patients will be affected, which will easily lead to negative emotions in the patients and affect their interpersonal communication [19].

For malocclusion, orthodontic treatment is the main treatment method. It mainly uses the external stress generated after the orthodontic device is worn to adjust the coordination between the patients’ maxillofacial, teeth and other bones and muscles, thereby correcting the abnormalities between the upper and lower teeth, between the upper and lower jaws, between the teeth and the jaws, which can promote the balance and stability of the stomatognathic system and improve the aesthetics of the maxillofacial [20–22]. The correction of patients with malocclusion during orthodontic treatment mainly relies on the aligners worn in the mouth. After wearing the aligners, it will exert force on the patients’ teeth, alveolar bone and jaw, making the teeth, alveolar bone, and jaw be shifted, thereby improving the crowding of the dentition.

The aligners used in orthodontic treatment are the key to ensure the effective correction of malocclusion by orthodontic treatment. The choice of aligners is related to the effects of orthodontic treatment on malocclusion. In the past, the orthodontic appliances used clinically for patients with malocclusion were mainly straight wire appliance. This appliance is fixed and worn in the patients’ mouth. Among them, the metal brackets and wires can exert forces in different directions and sizes, and can promote the movement and adjustment of the teeth by exerting continuous slight pressure on the teeth, thereby correcting the malocclusion to a certain extent. However, due to the limitation of insufficient aesthetics when wearing traditional fixed appliances, acceptance of many adult patients with malocclusion to this fixed appliance is not high and they are not satisfied during receiving orthodontic treatment.

In recent years, in order to improve the aesthetics of the aligners during orthodontic treatment, the clear aligners without brackets have gradually been used clinically to carry out orthodontic treatment for patients with malocclusion [23]. Clear aligners without brackets is a commonly used orthodontic the clear aligners without brackets, mainly through computer-aided design and production, using hot-pressed film technology to make the resilience generated after deformation to promote tooth displacement, so as to correct the irregularity of the tooth. In order to investigate the effects of non-bracket clear aligners on patients with malocclusion after orthodontic treatment, this study carried out a retrospective study on two groups of malocclusion patients who applied traditional fixed aligners and non-bracket clear aligners. After analysis and comparison, it was found that the total effective ratio was compared between the 2 groups, and the total effective ratio in the research one was greater (*P* < 0.05); after therapy, the measured values of the three periodontal status indicators of the plaque index, debris index, the gingival bleeding index were less than the control one; the measured values of mastication efficiency in the research one were greater than the control one; the measured values of serum inflammatory factors of CRP, IL-6, TNF-α were less than the control one (all *P* < 0.05), indicating that the orthodontic treatment effects of non-bracket clear aligners on patients with malocclusion are better than that of traditional fixed aligners, which can better improve the periodontal health of sufferers, decrease the periodontal inflammatory reactions, so that the patients’ masticatory function can be effectively restored. This research also found that after therapy, the research one had greater scores in the evaluation of comfort than the control one, and the two points in the psychological evaluation of the research one were less than the control one. The study group had better values in latency to fall asleep and actual sleep duration, and lower score in assessment of sleep quality. The scores obtained by the study group in the four dimensions of life quality evaluation were greater than the control one (all *P* < 0.05), indicating that clear aligners without brackets can also improve patients’ comfort, psychology, sleep, quality of life and other aspects. The reason is that the clear aligners without brackets can improve the periodontal health of sufferers and better correct dentition deformity. On the other hand, compared with traditional fixed aligners, the clear aligners without brackets are more beautiful after wearing, which can make patients more satisfied with them, and then help alleviate the negative emotions of patients due to insufficient aesthetics, thereby reducing adverse effects on their sleep and life quality.

Due to the small sample size, the analysis of the results is limited, and as this study is a retrospective study, it is not possible to further analyze the psychology of the study subjects from the perspectives of gender, psychology, and social roles. Alrwuili [24] showed in the study that pain is an important obstacle in receiving orthodontic treatment, which can reduce patient compliance with treatment. However, this study did not conduct a more in-depth analysis on this.

## Conclusions

In summary, in the orthodontic therapy of sufferers with malocclusion, compared with traditional fixed appliances, the clear aligners without brackets can enhance the treatment effects, and could have a good function in improving the periodontal condition such as plaque index, debris index, and gingival bleeding index, and masticatory function, and can reduce the inflammatory factors (CRP, IL-6 and TNF-α), which can make patients feel more comfortable, and improve their psychology, sleep and quality of life.

## Data Availability

The data that support the findings of this study are available from the corresponding author, upon reasonable request.
